# Water Buffalo’s Adaptability to Different Environments and Farming Systems: A Review

**DOI:** 10.3390/ani15111538

**Published:** 2025-05-24

**Authors:** Antonella Chiariotti, Antonio Borghese, Carlo Boselli, Vittoria Lucia Barile

**Affiliations:** 1 Council for Agricultural Research and Economics (CREA)—Research Centre for Animal Production and Aquaculture, via Salaria 31, 00015 Monterotondo, Italy; vittorialucia.barile@crea.gov.it; 2 General Secretariat International Buffalo Federation, via Salaria 31, 00015 Monterotondo, Italy; antonio.borghese@email.it; 3 Experimental Zooprophylactic Institute Lazio and Toscana “Mariano Aleandri” (IZS), National Reference Center for Ovine and Caprine Milk and Dairy Products Quality (C.Re. L.D.O.C.), via Appia Nuova 1411, 00178 Rome, Italy; carlo.boselli@izslt.it

**Keywords:** buffalo, extensive farming, family farming, humid savannah, intensive farming, marshlands

## Abstract

The buffalo species (*Bubalus bubalis*) is crucial for the global economy, supplying high-nutritional-value animal proteins vital for children’s growth. These animals efficiently convert fiber into energy and thrive in various harsh environments, from frigid climates to hot, humid areas, including wetlands. They produce milk and meat while supporting the sustainability of ecosystems that other ruminants cannot inhabit. Buffalo offers a unique opportunity to supply resources for both rural communities and larger farms located in specific regions, such as marshlands and humid savannahs. They also thrive on extensive pastures and family farms, thus preserving biodiversity, habitats, and cultural practices. Intensive farming brings distinct challenges and is often criticized for its negative effects on climate change. To counter these impacts, multiple strategies have been researched and implemented. These include enhancing livestock genetics, adopting sustainable agricultural practices, optimizing local feed resources (including by-products), managing manure (with an emphasis on renewable energy), and improving animal health and welfare. This review explores various buffalo farming system applications in different global contexts. It is based on the hypothesis that the adaptable traits of buffalo, as well as the environmental and economic challenges that must be addressed for sustainability, are the key factors in determining the viability of such enterprises.

## Simple Summary

Water buffaloes are highly adaptable animals that thrive in various farming systems worldwide—extensive, family, and intensive—with each impacting sustainability, biodiversity, and production efficiency. Extensive farming relies on grazing pastures with diverse plant communities, balancing herbivore populations to ensure consumption aligns with plant productivity. Family farming is vital for global food security and sustainable resource management. Despite challenges such as limited veterinary services, it remains eco-friendly and culturally significant, preserving biodiversity and local traditions, especially in regions like Asia, where small-scale mixed-crop–livestock systems utilize water buffalo as agricultural labor and financial reserves. Intensive farming seeks improved productivity through better feed efficiency and advanced management, driven by population growth and urbanization. However, it raises concerns over environmental impacts and animal welfare, underscoring the need for sustainable practices that respect animal behaviors. The integration of smart farming technologies is promising for enhancing water buffalo management. Overall, despite the advantages of these systems, challenges like disease management, climate change, and economic factors must be tackled for buffalo farming to be sustainable. This review explores various water buffalo farming system applications in different global contexts.

## 1. Introduction

There is a growing concern that the demand for animal products, associated with population growth, prolonged lifespan, and improved economic welfare, particularly in developing countries, will put unsustainable strain on the environment [1].

Global agriculture is responsible for between 8% and 18% of total anthropogenic greenhouse gas (GHG) emissions. The livestock sector is a significant contributor to these emissions, primarily through enteric fermentation; the production of manure, methane, and nitrous oxide; and various manure management systems that generate different levels of emissions [2].

According to Leroy et al. [3], the significant heterogeneity inherent in animal-derived food production, which encompasses categories such as dairy and meat and eggs, must be acknowledged. These products are characterized by distinct production methodologies and biochemical and nutritional properties, particularly within the context of varying ecological regions, resulting in diverse dietary requirements amongst population groups. Consequently, the consumption of animal-sourced foods varies considerably between geographical areas and socio-economic categories.

In the general debate, the complexity of the food system is often neglected and reduced to three interconnected claims that consumption of animal-sourced foods causes harm to human health, the planet, and the animal itself, related to health hazards, climate change, and animal welfare, forgetting that livestock sustains the livelihood of millions of people in the world (up to 12%), both in developing and developed countries [3].

Water buffalo (*Bubalus bubalis*) is renowned for its adaptability, which renders it pivotal to a wide range of production systems, encompassing both family farms and intensive livestock enterprises. It demonstrates an exceptional capacity to function efficaciously in diverse and occasionally extreme climates [4]. In addition to providing high-quality milk and meat [5,6,7], buffalo species play an essential role as work animals in daily agricultural operations, contributing to soil fertilization through their manure.

The present review aims to explore the adaptive capabilities of water buffalo in various agricultural settings, emphasizing their significant impact on rural economies and the environment. The review highlights the necessity of adopting sustainable and innovative farming practices, which are imperative in addressing the challenges posed by climate change and resource management.

Through in-depth analysis, the review aims to highlight the potential and the issues related to buffalo production in different environments and farming systems, challenging conventions and promoting a more sustainable approach that can also ensure the preservation of biodiversity and cultural traditions in rural communities.

## 2. Buffalo Population and Characteristics

The global buffalo population was estimated to be 209.1 million in 2023 [8]. The vast majority of the population (98%) is concentrated in Asia, predominantly in India and Pakistan, where breeds specifically selected for milk production are prevalent. In the African context, the distribution of buffalo is limited to Egypt, where the species is recognized as a valuable genetic resource and serves as a pivotal dairy animal for smallholder farmers. In addition, buffalo has considerable potential for development in South America, due to the abundance of grassland and free pasture and to the adaptability of the species to marshland and savannah. Most of the European buffalo population is located in Italy, a development that can be attributed to the expansion of the market for mozzarella and other cheeses [4].

The species plays a strategic role in the global economy and society, with an annual production of 150.3 million tons of milk, 98.9% of the total produced in Asia, and 7.09 million tons of meat, 96.5% produced in Asia. The production of milk and meat from buffalo herds has increased significantly in recent years, driven by either population growth or genetic improvement; between 2012 and 2022, the milk yield increased by 27% and meat yield by 14% [8].

It should be emphasized that these qualities are concomitant with high-quality buffalo products. Buffalo meat has a lower calorie content, lower cholesterol, an unsaturated fatty acid/saturated fatty acid ratio of >0.45, higher protein content, and a higher iron content (>1.5 mg/100 g) compared to beef. Buffalo milk is richer than cow’s milk in all major components, such as fat (6.6–8.8%), lactose (4.5–5.2%), protein (3.8–4.5%), casein, and ash, and it is often associated with a lower somatic cell count [9]. These chemical characteristics allow twice the cheese yield than that normally obtained with cow’s milk. Furthermore, the A2 versus A1 variant of β-casein makes this milk more similar to human breast milk, and it is probably easier to digest [10].

The milking technique may influence milk quality. Buffaloes are still milked by hand in many parts of the world, especially in Asia, where family farming is widespread, even if the first milking machine was applied more than 75 years ago [11]. The limitations of hand-milking compared to mechanical milking are the poor milk hygiene, the higher labor costs, and the number of buffaloes milked per milker (12–15 buffaloes per milking session). However, mechanical milking is not feasible in marginal areas where there is no electricity or in a herd with few animals (1–2) [12].

Buffalo exhibits numerous advantageous characteristics in comparison to other ruminants [4]. The capacity for conversion of fiber into energy is a primary factor in buffalo farming exploitation. Several studies have demonstrated the superiority of buffalo over cattle in feed conversion and the capacity to use low-nutrient forages and agricultural by-products [13]. In addition, a recent molecular study suggests that buffalo rumen has less potential for gastrointestinal methane production than bovine rumen [14].

Other important characteristics are its longevity (often exceeding eight lactations), rusticity, and ability to adapt to different climates (from hot and humid to very cold), which are always greater than cattle [4].

The buffalo has demonstrated a remarkable capacity for adaptation to harsh environmental conditions. It has developed several mechanisms to cope with extreme temperatures, including morphological, physiological, and behavioral adaptations. The buffalo epidermis is notably thick and rich in melanin, a trait that is critical for thermoregulatory functions [15]. The darker pigmentation of their skin and the absence of a dense hair coat, along with a reduced number of sweat glands, can result in a diminished heat dissipation capacity under these conditions, resolved through the habits of wallowing and searching for shade [16].

Moreover, the buffalo plays a vital role in the maintenance of clean riverbeds and wetlands. The large size of the buffalo’s hoofs renders it well-suited for agricultural activities and the maintenance of wetlands, including during the process of wallowing [4]. A notable characteristic of the species is its capacity to extirpate weeds, a function that is especially pertinent during the rainy season when the muddy ground hinders the mobility of other species. This ecological function is pivotal in preserving the integrity of water quality and biodiversity within these ecosystems. The buffalo can enter and work in rice fields, particularly in terraced areas, where mechanization is not an option and cattle cannot survive. These characteristics have made the buffalo the best draft animal in many countries, especially the swamp type [4].

Despite their advantageous characteristics, buffalo production remains smaller than bovine production due to lower milk production and growth rates, longer calving intervals, and the challenges in breeding and management. Buffalo calves have slower growth rates than bovine calves, leading to a longer time to reach market weight. The dressing yield is lower than that of cattle due to a higher proportion of non-carcass components like hide and head [17]. In some regions, there may be a preference for beef over buffalo meat, influencing consumer demand.

## 3. Farming Management Systems

Due to its peculiar characteristics, as discussed in Section 2, buffalo can adapt to different environments and management systems. These include (a) extensive farming, including wetlands and humid savannah, (b) family farming, and (c) intensive farming.

Nevertheless, adopting optimal sustainable farming systems is not straightforward, as they may yield different outcomes. In some instances, they may favor biodiversity conservation and C sequestration, whereas in others, they may prioritize production. For example, grazing-based systems may exhibit superior environmental performance due to the reduced input required for production, although they use more land. However, when land use options are incorporated into the assessment methodology, the results on environmental impacts may vary. Thomassen et al.’s [18] life cycle assessment (LCA) results showed lower land use per kilogram of conventional milk compared with organic milk.

### 3.1. Extensive Farming

The extensive farming system is based on grazing pasture. A natural pasture is a system characterized by a mixed botanical composition, including plant communities in which grasses (*Poaceae*) are the dominant species, with dicotyledonous herbaceous class (*Magnoliopsida*) present in varying proportions. An improved pasture is one sown with selected species [19]. Furthermore, following the agroecological conditions of the climate, soil fertility, grazing pressure, pests, and livestock needs, there could be associations with varying legumes, weeds, trees, and shrubs. The presence of trees, which defines the agropastoral or agrosilvopastoral systems, influences the growth, morphology, and nutritional value of forage, affecting climatic conditions; these include shading, air temperature, relative humidity, wind speed, and changes in soil water dynamics and nutrient cycling [20,21].

In the wake of global societal expectations to better balance livestock production and reduce GHG emissions [22], integrated crop-livestock-forest systems appear to be an evolution of mono-pastoralism and a positive alternative [23]. When silvopastoral systems are associated with intensively rotated pastures, they have a higher productive index with a stocking rate of up to 3.2 livestock units/ha/year, providing animal shading. Moreover, the meat from silvopastoral systems tended to have a lower ratio of omega-6/omega-3 fatty acids (ω6:ω3), which is more beneficial to human health. A lower ratio is more desirable for reducing the risk of many of the chronic diseases, including cardiovascular disease, cancer, and inflammatory and autoimmune diseases [24].

In the view of Casimir and Rao [25], a pasture is sustainable if there is a balance between the first trophic level (pastures), the consumers of the second trophic level (herbivores), and the consumers of the third trophic level (humans). This balance is maintained by ensuring that the population densities of both plants and herbivores remain relatively constant while consumption does not exceed the primary productivity of plants. Similarly, a sustainable pasture should consider practices that balance production objectives with social values and ecological needs [26]. Furthermore, permanent grasslands play an essential role in climate stability, as they store a comparable amount of C to that stored by forests [27]. Additionally, the manure produced by grazing animals has the potential to mitigate the risk of desertification, thereby improving soil functionality in terms of structure, organic matter content, and resilience to erosion by wind or water. By adding organic matter, manure increases soil aggregation, improves water infiltration and retention, and enhances soil stability, making it less susceptible to wind and water erosion [28]. When handling and spreading manure, no significant amount of methane is added when spread directly on pastures and fields [29]. Grazing animals, through their manure and grazing patterns, can further contribute to these benefits.

It has been indicated by several authors that the efficient management of a pasture can guarantee economic sustainability, leading to the elimination of fixed costs, such as those associated with the construction of a barn, and the reduction in variable costs, including the direct collection of feed from natural pastures by the animals themselves [28,29]. Nevertheless, the findings of other researchers did not demonstrate a substantial impact of the grazing type on the economic efficiency component [30].

In grazing-based systems, forage intake, animal behavior, and performance may be hindered by various factors. These include heat stress, which can impact animal health [30], and the low availability and nutritional value of forage, such as high proportions of stems or dead plant matter, may deter animals from foraging [31]. The consequences of this may be reduced productivity and lower milk yields [32].

Moreover, grazing should be properly streamlined to avoid the generally claimed excessive ecological damage caused by animal overgrazing, trampling, soil compaction, and overuse of water sources [32]. In the case of buffalo, the causes of these impacts can be ascribed to thermoregulation habits, including wallowing in the mud, especially in periods and areas characterized by hot climates [33].

Extensive farming practices may reduce both environmental impact and costs while promoting animal welfare and quality product differentiation [34]. Such practices may be conveniently employed for species well adapted to the environment, such as buffalo. In Italy, where buffalo is reared intensively, Sabia et al. [35] reported that adopting a free-ranging system for non-productive categories, such as heifers, may reduce water consumption and most polluting agents in the atmosphere and water. They observed a reduction in the impact of climate change (9%), terrestrial acidification (10%), marine eutrophication (6%), and water depletion (11%), whereas agricultural land occupation was 7% higher.

**Figure 1:**
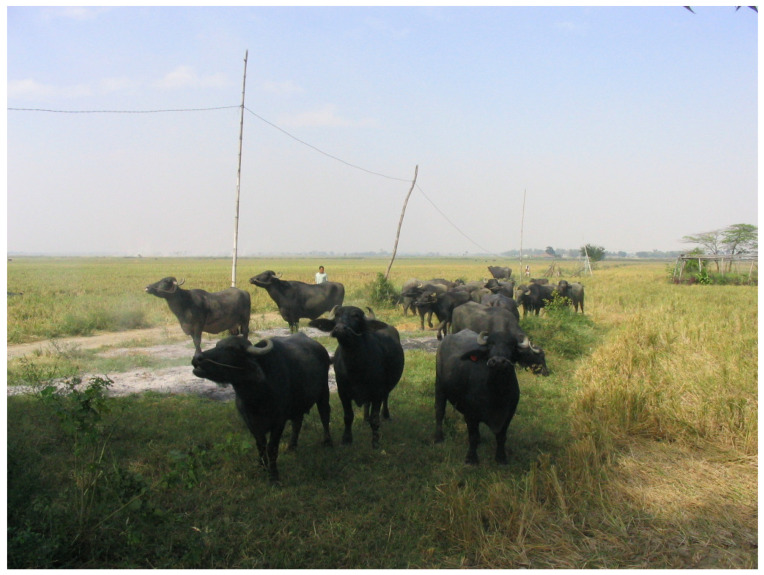
An example of water buffaloes raised in an extensive system in the Philippines (photo: Barile).

**Figure 2:**
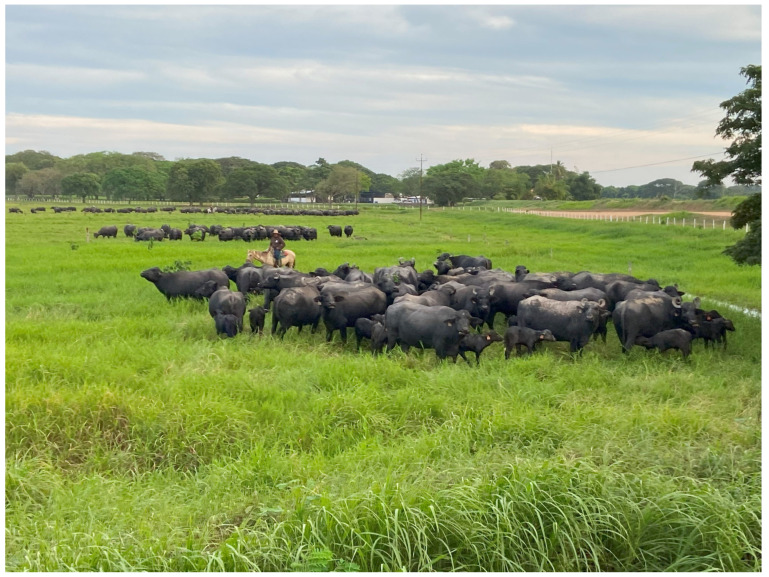
An example of water buffaloes raised in an extensive system in South America (photo: Chiariotti).

The availability of a great extension of land, rich in pasture, in some areas in Asia (e.g., Sumatra, Sri Lanka, and Philippines—Figure 1), Australia, and much more in Central and South America (e.g., Brazil, Argentina, Venezuela, and Paraguay) represent a good opportunity to realize a new economy based on grazing (Figure 2) [4].

In Brazil, the country with the largest number (1.39 Mil head) [36] and the most developed industry in South America, buffalo is produced in the following four different grazing ecosystems in the Amazon region: (a) native flooded pastures on the island of Marajó; (b) native flooded pastures in the lower and middle Amazon; (c) native dryland pastures; and (d) cultivated dryland pastures, in areas previously used for other agricultural purposes, either in monocultures or in integrated systems that deliberately combine pastures, trees, and animals in the same area and at the same time. In this latter ecosystem, forage grasses are highly productive and nutritious, with an average crude protein content of 13.3% and an in vitro organic matter digestibility of 52.3% [37]. This highly fertile land is flooded for 6 months a year, from January to July, with sedimentary deposits from the Amazon River and its tributaries. The minerals in these forage grasses are sufficient for the maintenance and production needs of the animals. Therefore, the main problem is the flooding itself.

The majority of the buffalo reared extensively are in humid, tropical, or subtropical wetlands, where they are far superior to the bovine breed in productive efficiency.

Wetlands are present in different world regions, including marshlands in Iraq, Bangladesh, and Indonesia, as well as humid savannahs in Venezuela, Brazil, Argentina, Paraguay, and Colombia. The estimated area of the world’s wetlands is between 7 and 9 million km^2^, representing a significant proportion of the planet’s terrestrial surface [38].

Marshes are wetlands saturated in water and composed mainly of grass and reeds near the fringes of lakes and streams, serving as transitional areas between land and aquatic ecosystems. Their ecosystems are distinguished by hydrology, physicochemical environment, and biota, and play an essential role in the evolution of life on Earth. Historical evidence demonstrates that these ecosystems have been vital for the survival of human communities [39].

Several strategies can be adopted to maintain water quality in the context of livestock management in marshland areas. These include the prevention of excreta accumulation in bodies of water and the banning of herbicide use. Additionally, the reduction in landscape modifications that affect the hydrological regime and water quality, such as the construction of dams or canals, is of utmost importance. Furthermore, the rotation of livestock distribution areas can help minimize nutrient input, and the rational use of veterinary products can help mitigate the impact of these products on the environment and water quality.

**Figure 3:**
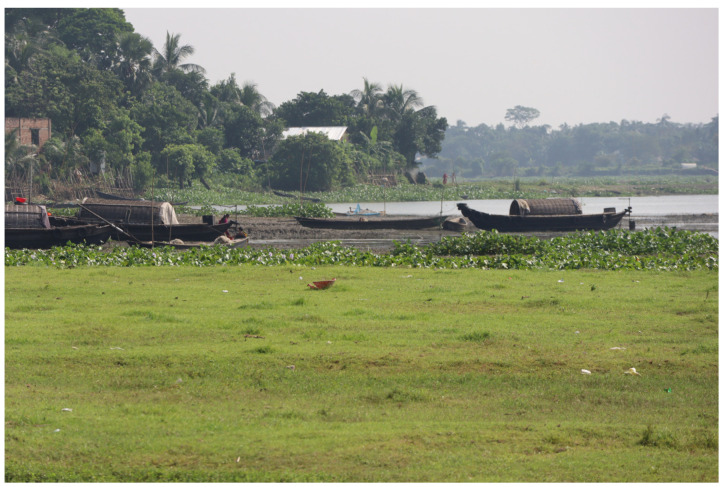
Examples of wetlands. Marshland in Bangladesh (photo: Chiariotti).

An example of marshland is the *bathan* in Bangladesh, in the saline coastal region. *Bathan* is a type of agrarian business that flourished when livestock represented a crucial factor in the rural economy and social relations. The *bathania* used to maintain cattle of the village community in the field and in return, received remuneration in kind from the community. *Bathan* is still noticeable in some zones of Bangladesh, where temporary settlements are formed in the winter in the *Haor* beds and dismantled when the monsoon comes. The buffalo in household subsistence farming and extensive *Bathan* farming are used as draft animals and partially for milk and meat production [40] (Figure 3).

In Iraq, buffalo breeding occurs in the marshes and along the Tigris and Euphrates rivers, where they swim extensively for sustenance, consuming papyrus, reeds, and other vegetation. During periods of high floodwater, the owners collect these plants to feed the buffaloes on platforms [4].

**Figure 4:**
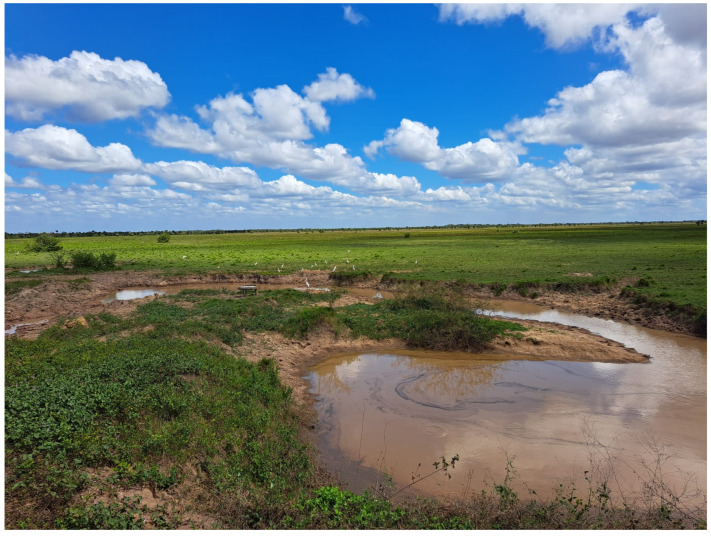
Examples of South America’s wetlands. Venezuelan savannah (Chiariotti, photo).

Among the wetlands, savannah is the terrestrial biome, mainly subtropical and tropical, found in numerous transition zones between rainforest and desert or steppe, mainly in Central and South America (Figure 4), Central Africa, India, Indochina, and Australia.

The vegetation is predominantly shrubs and trees, arranged in a way that does not result in the formation of a closed canopy. Tropical and subtropical savannah distribution is determined by rainfall scarcity and marked seasonality. Rainfall of less than 2000 mm per year is insufficient for developing trees and shrubs, resulting in grassland formation. As one progresses toward more humid latitudes, specifically toward the equator, one observes the emergence of shrub vegetation (up to 3000 mm of rainfall). Grass grows more rapidly in wet months and desiccates during the dry season.

It has been demonstrated that the tropical savannah has evolved in conjunction with grazing, resulting in modifications to the structural and functional components of the ecosystem [41]. This is achieved through vegetation clipping, trampling on soil, dunging, and urination [42]. Soil properties are known to change due to compaction, which in turn affects soil organic C and nitrogen (N) levels [42]. The impact of livestock trampling on soil properties is further intensified when current grazing levels in most savannahs are unchecked [43]. Grazing during drought will induce soil and plant water stress that will destabilize soil C and N contents, therefore affecting other soil properties and plant physiological processes. Increased rainfall beyond ambient is highly unlikely to affect most soil properties due to its minimal effect on soil moisture [44].

Buffalo is uniquely suited for the efficient harvesting of biomass and cleansing of riverbeds, thereby facilitating the regeneration of aquatic plants, seeds, and small organisms, which serve as sustenance for aquatic birds. It is evident that its presence significantly enhances the biodiversity and abundance of wetland species. In these environmental conditions, the buffalo may produce milk and meat with high nutritional value, thereby making the wetland farming system a compelling model for habitat conservation and a strategy against climate change [4].

### 3.2. Family Farming

Family farming is the most prevalent food and agricultural production system. The International Steering Committee for the International Year of Family Farming, launched by the Food and Agriculture Organization of the United Nations (FAO), in 2014, adopted the following definition of family farming: “a means of organizing agricultural, forestry, fisheries, pastoral, and aquaculture production which is managed and operated by a family and predominantly reliant on family labor, including both women’s and men’s”. The committee further states that “the family and the farm are linked, co-evolve, and combine economic, environmental, social, and cultural functions” [45].

The family and the farm are strongly interlinked; the family provides the farm’s labor force and resources [46]. Family farmers range from smallholders to medium-scale farmers and include peasants, Indigenous peoples, traditional communities, fisher folks, mountain farmers, pastoralists, and many other groups representing every region and biome of the world [47]. The role of family farmers in alleviating hunger and poverty, improving food security and nutrition, sustainably managing natural resources, and protecting the environment is well-documented [48,49,50].

Family farmers have been identified as key contributors to achieving many of the Sustainable Development Goals (SDGs), with the potential to guide us toward more productive and sustainable food systems [51]. Indeed, it is estimated that over 90% of farms are run by an individual or a family, with women providing almost 50% of farm labor and holding less than 15% of farmland; their role is particularly important in the production, processing, and marketing of dairy products. Furthermore, family farms are responsible for over 80% of the world’s food production, managing around 70–80% of farmland worldwide [45].

In addition to declining employment and rural depopulation, family farming is a more environmentally friendly, less external input-dependent, regionalized production system. This may require a reversal of the global trend toward increasing specialization for a recoupling of crop and livestock production, not least for the resilience it provides. Alongside this trend toward scale, small farms persist in Asia; farm consolidation is moving at a snail’s pace in Southeast Asia, and 70% of farms in India are “ultra-small”—less than 0.05 ha [52].

Several general issues are associated with family farming for buffalo management in many developing countries (Thailand, Iran, Nepal, Bangladesh, Sri Lanka, Vietnam, Laos, Malaysia, Philippines, Cambodia, China, India, Pakistan, and Egypt). These include the lack of housing systems, the absence of artificial insemination, routine vaccination programs, and animal identification and recording systems. The small farms as often scattered in remote areas or do not have the economic means to access veterinary services. It is imperative to integrate genetic enhancement programs with other livestock development initiatives, including health services, nutrition, management, and access to output markets in smallholder production.

In various Asian countries, buffalo are kept by smallholder farmers in mixed crop-livestock systems, which are primarily practiced to generate adequate income, provide food security for family members and manage and conserve natural resources for sustainable agricultural production. Moreover, the animals provide a crucial source of draft power, the manure produced by the buffalo serves as a natural fertilizer, and last but not least, the buffalo themselves act as a financial reserve, providing a safety net in times of economic hardship. In the event of financial distress, the farmers may opt to sell their livestock to address any shortfalls in their income.

Most livestock farms are small-scale in terms of farmed area and herd size, with an average number of <20 heads, and generally include daytime grazing (field margins, roadsides, marginal land, communal grazing areas, etc.) and are supplemented in stalls [53].

**Figure 5:**
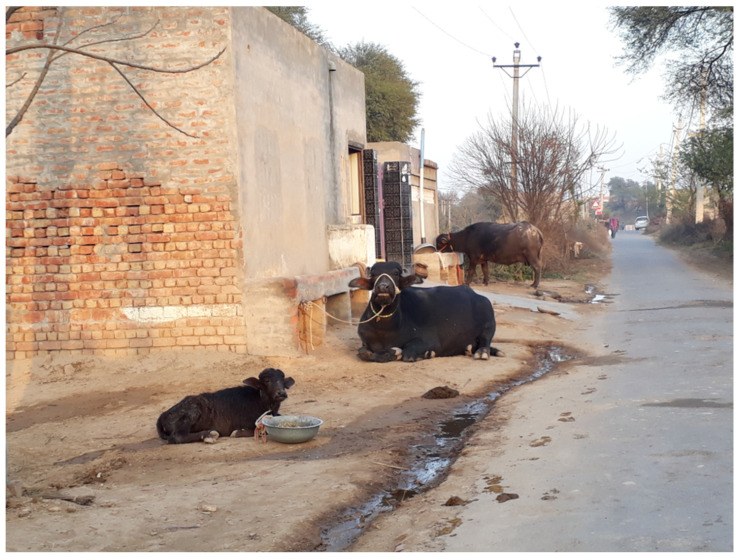
An example of water buffaloes reared in family farming in India. The animals are kept near the household (photo: Chiariotti).

In India, depending on the number of buffaloes owned and the land and fodder resources available, small farmers keep their buffaloes in two types of management. An extensive system with one to two buffaloes maintained on natural grasses on communal land, supplemented with agricultural by-products using family labor and traditional technology (Figure 5); or in a semi-intensive system with three to five buffaloes maintained on irrigated fodder, crop by-products, and concentrates, with improved housing and care. In recent years, with the availability of efficient breeding services and a good market for milk, smallholder farmers are seeking to feed their high-yielding buffaloes well, and many of these farmers are dedicating their small holdings or leasing land from others to grow good-quality fodder [52].

In some Asian countries, buffalo and cattle coexist on a single farm, as in Nepal, where 58% are smallholders, but buffalo is becoming popular because of its adaptability to local feed resources and the higher milk price [53].

In Egypt, 92% of farmers, holding 88% of the livestock, operate on small farms. Farms with less than 2 *feddan* (1 *feddan* = 0.42 ha) compose about 80% of all farms [54]. Most farms are mixed farms where livestock and crop production are integrated. Buffaloes are traditionally managed, hand-milked, and naturally bred, and farmers, subsequently, sell surplus raw milk and dairy products through informal markets [55]. Access to veterinary and extension services is inadequate and inefficient.

It is important to acknowledge that family farms perform a multitude of functions that extend beyond the mere production of food. These elements concurrently fulfil environmental, social, and cultural functions, thereby aiding in the mitigation of the risks associated with biodiversity loss, while simultaneously preserving the landscape and maintaining community and cultural heritage. Moreover, they possess the knowledge required to produce nutritious and culturally appropriate food as part of local traditions [45].

### 3.3. Intensive Farming

In its most basic definition, agricultural intensification can be understood as an increase in the production of commodities per unit of input, such as labor, land use, fertilizers, time, feed, animals, or capital [56]. Given that land is the ultimate limiting factor for agricultural production, it can be observed that most studies defining agricultural intensification focus on the increased output per unit area of land [57].

The primary drivers of livestock intensification are population growth, an increase in gross domestic product (GDP), urbanization, and the globalization of markets, all of which have collectively elevated consumer demand for animal products. This has led to a shift in focus within the livestock sector toward product intensification, accompanied by changes in branch industries and transportation, in developed and developing countries (i.e., Italy, India, and Pakistan).

The handling of buffaloes reared in intensive systems is comparable to that of cattle. The enhancement of feed conversion efficiency was the primary factor contributing to the intensification of livestock production per head. The intensification per hectare was predominantly ascribed to the elevated productivity per animal, the diminished rates of culling and replacement, and the increased forage and crop yields per hectare [58]. However, the expansion of intensive dairy systems inevitably results in heightened environmental impacts per hectare due to the increased incorporation of corn silage and concentrate feed into the diet, involving a greater input utilization. Consequently, it is challenging to reconcile environmental impacts with productivity in dairy systems [58].

In India and Pakistan, several large farms are expanding and generating growth in the dairy sector, which is highly significant in the countries’ economies, to supply food in urban and peri-urban areas. Intensive commercial farming is seen as the future of buffalo farming in these countries, driven by the growing demand and the necessity to improve farmers’ livelihoods [53]. The same tendency, even if on a smaller scale, is shown in Nepal, Bangladesh, China, and Thailand.

Intensive farms employ advanced breeding management techniques and mechanized equipment for daily operations. An example is the adoption of mechanical milking (from the simple bucket machine to the complex automatic milking system), widely recommended for milk yield, safety, and quality. Monitoring the milking routine and milking machines’ operating parameters is a crucial factor in ensuring optimal milk extraction, labor efficiency, and udder health in dairy animals [59]. Moreover, it should be noted that these farms require considerable capital investment and skilled operational management [53].

**Figure 6:**
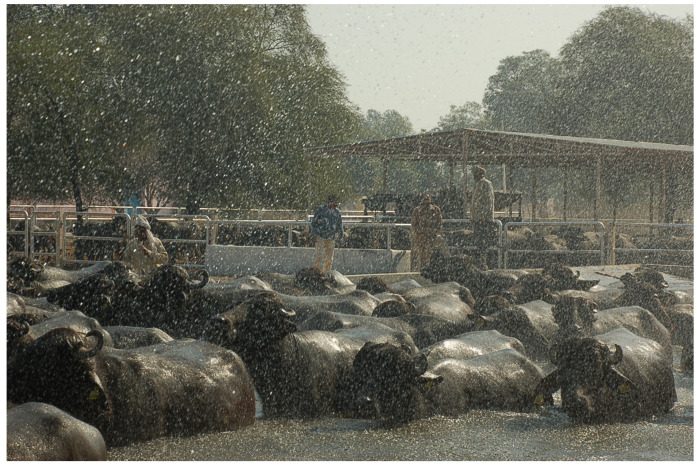
An example of intensive water buffalo farming in India. Refreshment system with pool and showers (photo: Chiariotti).

However, the implementation of the intensive system has given rise to some new stressors including artificial rearing of calves and reduction in space, which can affect health, social behavior, and heat dissipation, particularly when there is no access to pasture and water for wallowing [34] or pools or showers (Figure 6). Sustainability and animal welfare could also affect consumer preferences [60].

**Figure 7:**
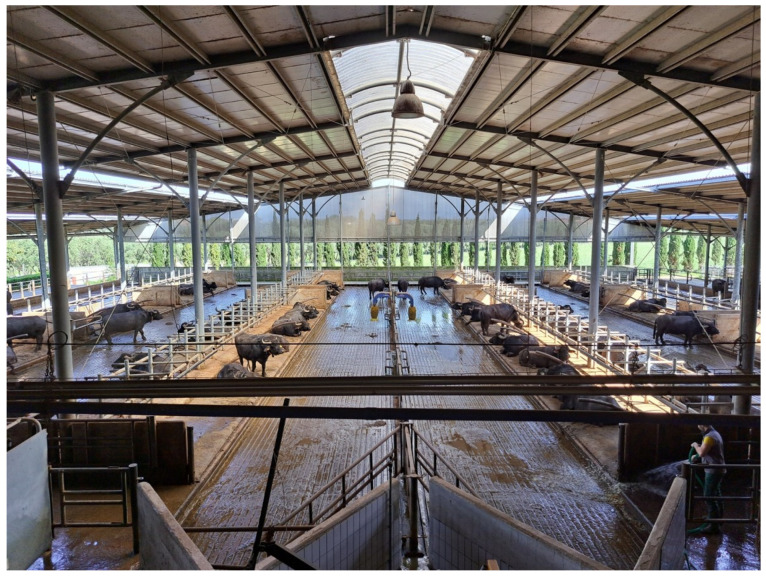
An example of advanced intensive farming management in Italy (photo: Barile).

In Europe, there are the following two opposite situations: countries which have a long tradition of buffalo breeding (Italy) and countries where buffalo has been newly introduced as a sustainable alternative to dairy cows (Germany, UK, and Greece). In Italy, where buffalo are intensively reared as dairy cattle, buffalo milk is not used for fresh consumption, but the entire production is processed into cheese, particularly *mozzarella (*Figure 7). The increased demand for this cheese, both in national and international markets, has favored increases in the buffalo population and production [4]. The consumption of buffalo meat within Italy is not in great demand and confined to local consumption in areas where buffalo farming is traditional, and the marketing of the product focuses on fresh meat cuts and delicacies, such as salami and bresaola [4].

It was more than two decades ago that Italy became a pioneer in robotic milking by adopting an advanced, intensive milking system (Milking Robot) for buffalo. The animals are permitted to be milked at their discretion (with an average of 2.5 milkings per day), thereby enhancing their welfare and increasing the milk yield for the farmer. According to the work of Boselli and Borghese [59], the effects include the facilitation of pre-stimulation, enhanced yield and quality of milk, augmented system capacity (defined as the number of buffaloes milked per year), improved milking hygiene, and, as a consequence, reduction in the incidence of mastitis.

Recently, some advanced techniques, such as smart farming, have been developed to increase the sustainability of intensive management. The smart farming system is a new concept in the agricultural field. It means managing farms using sensors, software, data analysis, communication systems, etc., enabling farmers to provide plants/animals with the precise treatment they need. Regarding livestock, most of these technologies are utilized in the dairy cattle sector, where the use of automated and robotic devices is increasing rapidly.

In Italy, precision livestock farming (PLF) technologies have recently been applied to buffalo species to improve some critical aspects of the farm, such as nutrition, reproduction, and management [60]. To this end, ear-tag sensor systems are being tested on buffalo to record activity, rest, rumination, and temperature at the ear level [61]. This novel farm concept involves the integration of animals, environment, machinery and processes as “information objects” to augment data. Farm management and animal husbandry are conceptualized as systems of complexity, individual variability, temporal variability and dynamic nature (CITD) [60].

## 4. Future Perspectives

The concept of sustainable buffalo farming must consider several economic and social factors, including food security, poverty alleviation, mitigation strategies, and social and cultural value preservation. This can only be achieved by implementing policy initiatives involving all stakeholders in the supply chain. To address the impacts of climate change, it is essential to adopt sustainable agricultural practices, utilize local feed resources more effectively, and enhance the nutritional value of the produced food, considering land and water scarcity.

The buffalo’s genetic diversity and adaptability to different environments, including cold climates; hot, humid conditions; and wetlands where other ruminants cannot thrive, could contribute to the achievement of some of the SDGs set out in Agenda 2030.

Pastures are anthropogenic ecosystems that offer low-cost food resources, primarily for ruminant feeding, with buffalo playing a significant role in the production and livelihood of many rural communities. The intensification of buffalo meat and milk production in pasture has been demonstrated to be sustainable when rational grazing practices are employed [62,63]. In the future, crop–livestock–forest integration systems, including silvopastoral ones, will become increasingly relevant and help to reclaim agricultural areas that have been altered by inappropriate previous use. Such practices could potentially enhance the value of the land, facilitating more efficient nutrient cycling. Alternatively, they could improve animal welfare through natural shading or acting as a C sink.

Due to its anatomical and physiological characteristics, the buffalo is the only ruminant that can be reared in the wetlands. It can produce milk and meat of high nutritional value, making the marshland farming system an interesting model for habitat conservation. It can also increase biodiversity, which benefits the local population.

The family farming system is pivotal for sustaining people’s livelihoods. Moreover, utilizing local buffalo breeds, the most adapted to low-input production systems and specific environments, family farming helps preserve genetic biodiversity.

Future advances in PLF hold great promise, particularly in intensive production systems. Adapting to individual animal needs will help reduce inputs and improve animal welfare. Buffalo production systems, as with other livestock, are closely linked to sustainability, welfare, and product quality, and new technologies and training will play an important role in meeting the challenges of climate change.

## 5. Conclusions

Water buffalo is among the most resilient livestock species, capable of thriving in diverse environments and adaptable to different farming systems across the globe. From intensive dairy systems in Italy to extensive rice farming in Southeast Asia, it provides significant economic, social, and environmental benefits.

Buffalo’s multifunctional roles as milk producers, meat providers, draft animals, and ecosystem stewards in wetlands and family farms highlight their importance in sustaining rural livelihoods and the environment.

Challenges, such as disease management, economic fluctuations, and climate change, however, will require continued research and innovation to ensure the long-term sustainability of water buffalo farming worldwide.

## Data Availability

No new data were created or analyzed in this study.

## Abbreviations

Carbon	C
Complexity, Individual Variability, Temporal Variability, and Dynamic Nature	CITD
Food and Agriculture Organization of the United Nations	FAO
Greenhouse Gas	GHG
Gross Domestic Product	GDP
Life Cycle Assessment	LCA
Nitrogen	N
Precision Livestock Farming	PLF
Sustainable Development Goals	SDGs
